# Collaborative and individual learning of geography in immersive virtual reality: An effectiveness study

**DOI:** 10.1371/journal.pone.0276267

**Published:** 2022-10-18

**Authors:** Michal Sedlák, Čeněk Šašinka, Zdeněk Stachoň, Jiří Chmelík, Milan Doležal

**Affiliations:** 1 Department of Psychology, Faculty of Arts, Masaryk University, Brno, Czech Republic; 2 Department of Information and Library Studies, Faculty of Arts, Masaryk University, Brno, Czech Republic; 3 Department of Geography, Faculty of Science, Masaryk University, Brno, Czech Republic; 4 Department of Visual Computing, Faculty of Informatics, Masaryk University, Brno, Czech Republic; University of Milan, ITALY

## Abstract

Many university-taught courses moved to online form since the outbreak of the global pandemic of coronavirus disease (COVID-19). Distance learning has become broadly used as a result of the widely applied lockdowns, however, many students lack personal contact in the learning process. A classical web-based distance learning does not provide means for natural interpersonal interaction. The technology of *immersive virtual reality* (iVR) may mitigate this problem. Current research has been aimed mainly at specific instances of *collaborative immersive virtual environment* (CIVE) applications for learning. The fields utilizing iVR for knowledge construction and skills training with the use of spatial visualizations show promising results. The objective of this study was to assess the effectiveness of collaborative and individual use of iVR for learning geography, specifically training in hypsography. Furthermore, the study’s goals were to determine whether collaborative learning would be more effective and to investigate the key elements in which collaborative and individual learning were expected to differ–motivation and use of cognitive resources. The CIVE application developed at Masaryk University was utilized to train 80 participants in inferring conclusions from cartographic visualizations. The collaborative and individual experimental group underwent a research procedure consisting of a pretest, training in iVR, posttest, and questionnaires. A statistical comparison between the geography pretest and posttest for the individual learning showed a significant increase in the score (*p* = 0.024, *ES* = 0.128) and speed (*p* = 0.027, *ES* = 0.123), while for the collaborative learning, there was a significant increase in the score (*p*<0.001, *ES* = 0.333) but not in speed (*p* = 1.000, *ES* = 0.000). Thus, iVR as a medium proved to be an effective tool for learning geography. However, comparing the collaborative and individual learning showed no significant difference in the learning gain (*p* = 0.303, *ES* = 0.115), speed gain (*p* = 0.098, *ES* = 0.185), or performance motivation (*p* = 0.368, *ES* = 0.101). Nevertheless, the collaborative learning group had significantly higher use of cognitive resources (*p* = 0.046, *ES* = 0.223) than the individual learning group. The results were discussed in relation to the cognitive load theories, and future research directions for iVR learning were proposed.

## Introduction

The increasing amount of *immersive virtual reality* (iVR) technologies, applications, and research, since the emergence of consumer-oriented *head-mounted displays* (HMDs) in 2016, suggests that this technology will not only be a temporary trend but a new direction in human–computer interaction. It has been studied for its utilization in areas such as cognitive training [1, 2], psychotherapy [3, 4], rehabilitation [5, 6], pain relief [7, 8], laboratory safety training [9], museum exhibitions [10], heritage tourism [11], journalism [12], multidimensional data visualization [13], data analysis [14], urban planning [15], and education [16–19]. Our previous study [20] explored the educational use of iVR using qualitative methodology. In this follow-up study, we address the educational use of iVR using quantitative methodology and focus on two aspects–the suitability of iVR as a medium for education and the difference between collaborative and individual learning in iVR.

In the educational context, iVR offers the capacity for overcoming the limitations of a specific time and space, in a sense that education is not bounded by a classroom lesson at a specific hour, thanks to the possibility of synchronous and asynchronous learning in a virtual environment. To describe what iVR has to offer in the learning context, the concept of *affordance* formulated by Gibson [21] appears to be useful. It expresses an opportunity (option, possibility, or functional utility) that is offered by properties of a specific environment to a specific type of actors, defined by its characteristics. Shin [22] studied affordances that immersive virtual environments offer to people in the learning context. The identified affective affordances were presence and immersion, which had a significant influence on usability, which in turn had a significant influence on the educational affordances of empathy and embodiment. He argued that the learning process in iVR starts when the user perceives the virtual content with empathy and embodied cognition. To describe the process of learning in iVR, Makransky and Petersen [23] proposed the *Cognitive Affective Model of Immersive Learning* (CAMIL), which identifies the psychological affordances of presence and agency, and describes how they influence cognition and motivation. In the research by Meyer et al. [24], learning outcomes achieved with iVR or with video lessons were compared in groups with or without a pre-training, which consisted of a picture with main concepts that could be studied before the multimedia lesson to reduce cognitive load. The results showed that for the educational use of iVR, the pre-training had a significant impact on better knowledge retention and transfer, as well as on perceived self-efficacy. A review conducted by Jensen and Konradsen [25] identified that iVR is useful for skill acquisition related to visual and spatial information, however, stressed that more research is needed.

Immersive virtual reality applications have been emerging extensively in the field of geography, with the term *virtual geographic environment* (VGE) [26] often being used. Differences between 2D and 3D cartographic visualizations in iVR are discussed by Hruby et al. [27], focusing on their scale. They differentiate between downscaled 2D maps in iVR and a *geovisualization immersive virtual environment* (GeoIVE), which is considered to be a 1:1 scaled 3D geovisualization. Several studies on the usability of iVR for geography education have been conducted. In the research by Shakirova et al. [28], 60 students underwent geography training in two experimental groups–the first one using the traditional e-learning system, and the second one having the e-learning supplemented with iVR educational applications. Based on the self-assessment of the participants, the iVR-using group perceived a higher gain of professional knowledge, skills, and abilities. Moreover, the results of Klippel et al. [29] study, which compared the use of iVR for facilitating geoscience field trips with the use of traditional field trips in objective reality, showed advantages of iVR in students’ learning experience, lab scores, and enjoyment.

Social factors in learning have been getting much attention from researchers in recent years, with Hanko [30] summarizing their benefit as enhancing students’ enjoyment of learning and promoting their performance. She proposed establishing standards not only for student literacy but also for implementation of collaborative learning methods. Vygotsky’s concept of the *zone of proximal development* (ZPD) [31], describing how a student can advance their competences with support from a more competent teacher or collaborator, can be considered a predecessor to the newer approach of *scaffolding*. De Lisi [32] described it as a supportive framework in an educational process, which can include collaborative learning and computer-realized support. A relatively recent approach to collaborative learning called *computer-supported collaborative learning* (CSCL) [33] emerged from an intersection of cognitive psychology, informatics, and pedagogical theory. Stahl [34], however, stressed the importance of assessing whether the knowledge gained from CSCL would not also be gained in case of individual learning. Three types of affordances in the context of CSCL have been described by Kirschner et al. [35]: educational, social, and technological. Educational affordances are determined by characteristics of a collaborative learning paradigm and enable particular learning behavior. Social affordances are determined by characteristics of a group and enable particular social interactions. Finally, technological affordances are determined by properties of computer technology and enable particular actions and operations executable in a virtual environment. Herrera-Pavo [36] utilized these affordances in three case studies and stressed the importance of designing learning tasks to enable students to plan their work, evaluate their progress, and organize the solving process. Additionally, the research by Muñoz-Carril et al. [37] showed that students’ satisfaction in CSCL is significantly influenced by confirmation, perceived usefulness, and perceived enjoyment, the latter of which was studied through a theoretical framework of Csikszentmihalyi’s [38, 39] construct of *flow*, which is connected with intrinsic motivation.

The development of CSCL and its fusion with iVR technology led to the emergence of an even more novel approach called *virtual reality-based collaborative learning* (VRCL). According to Burdea and Coiffet [40], iVR is a powerful tool for learning due to its capacity to mediate collaboration with other users in a shared virtual environment, offering the opportunity to explore virtual objects and conduct experiments, as well as the possibility to assign roles to users. They stressed its potential to improve student motivation and knowledge retention, and its capacity to facilitate distance learning. Zheng et al. [41] identified three main affordances of iVR for collaborative learning as social interaction, resource sharing, and knowledge construction. The social interaction affordance highlights the support of iVR for discussion and collaboration in the learning process, providing a more intuitive and natural way for collaborative learning than traditional *non-immersive virtual reality* (non-iVR) collaboration. As for the resource sharing affordance, thanks to the representations that iVR offers, collaborators have an opportunity to share information in a more embodied manner and can see what each of them is currently working on. Lastly, the knowledge construction affordance highlights the scaffolding for complex and meaningful problem solving and high-level cognitive activities.

In our previous qualitative study [20], we utilized interpretative phenomenological analysis to describe the cognitive and social phenomena underlying the use of *collaborative immersive virtual environment* (CIVE) for education in geography. The results showed that the participants felt the benefit of iVR for learning geography and considered the spatial representation of terrain as helpful. They also appreciated the help of a collaborator in iVR and were glad they had some kind of personal contact. However, they lacked the visibility of emotions on the avatar in iVR and struggled with the limitations of iVR controllers. The study uncovered the themes significant for collaborative learning of geography in iVR, but the qualitative nature of the research had its limitations. It was not possible to objectively determine if the participants benefited from the iVR collaborative learning. Therefore, a follow-up quantitative study was designed to examine iVR as a medium for learning and the benefit of collaborative use. The objective of the present follow-up study was to quantitatively assess the effectiveness of individual and collaborative learning of geography in CIVE, and to determine whether the collaborative use is more effective. Furthermore, this study aimed to provide new insights into the key elements in which the individual and collaborative learning in CIVE differ, by investigating the use of cognitive resources and the motivation of users.

## Methods

### Materials and technology

**Fig 1:**
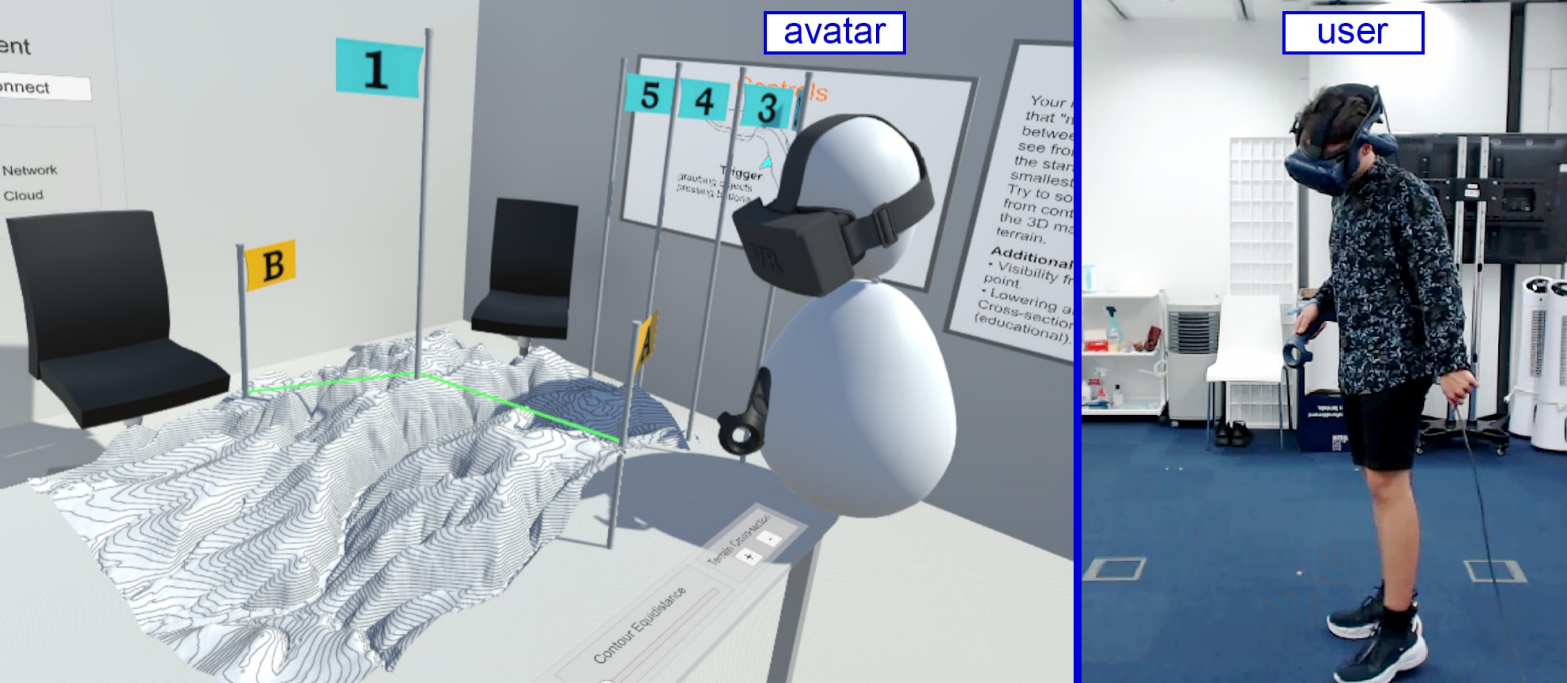
Geography learning CIVE application developed at Masaryk University.

This quantitative study utilized the geography learning CIVE application, developed by our interdisciplinary team, which was described in detail in our previous qualitative study [20]. The application enables users to solve geospatial tasks in an experimental environment, offering multiple options for visualization and tools for testing users’ hypotheses, as shown in Fig 1. It is based on the principles of *scientific discovery learning* (SDL) [42] and facilitates *learning through problem solving* (LPS) [43]. One of the major advantages of the application lies in the possibility of switching between 2D and 3D geovisualization displayed via a 3D imaging device of iVR. Thanks to that, the user can examine the 3D model from all sides and angles and develop an association between what the contour lines look like on a classical map and what they represent in the real-world terrain. Our application offers the scaffolding necessary for learning hypsography and can be used individually or collaboratively.

**Fig 2:**
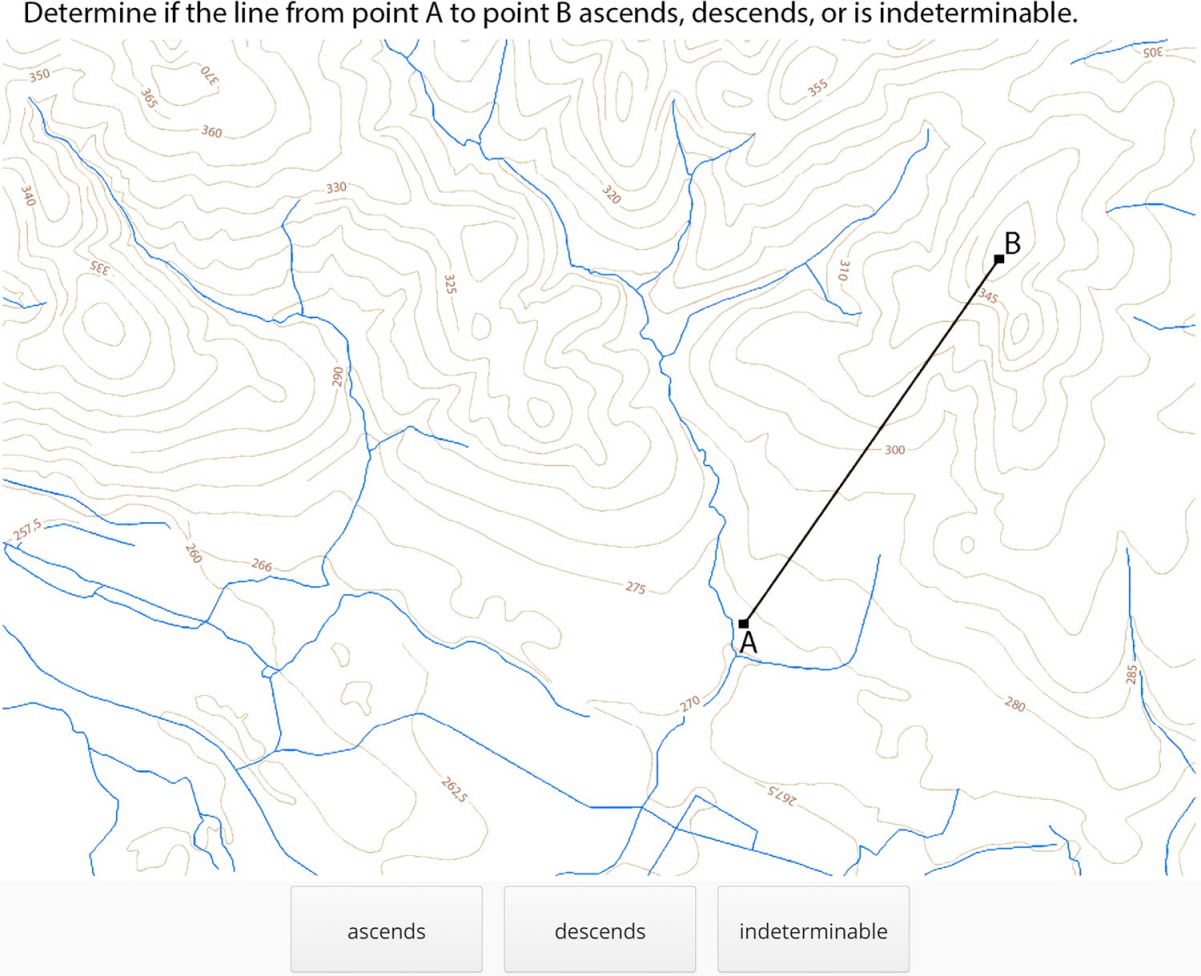
Test item created in the hypothesis platform for the pretest and posttest.

To test the level of users’ hypsography skills before and after educational training in the CIVE application, a pretest and posttest were created on the platform called Hypothesis. It enables the construction of cartographic and psychodiagnostic test batteries, which are stored on the servers of Masaryk University and can be administered on any computer connected to the internet [44]. Among the biggest advantages of this platform are the ability of objective automatic testing, precise reaction time measurement, and automatic results evaluation. To serve as the pretest and posttest, two geography tests named “geo α” and “geo β” were created. These focused on map reading and map analysis, which can be considered cognitive and declarative based on the classification of map skills [45]. Both of these consisted of four types of tasks: 1) Select the lowest/highest point; 2) Determine if there is direct visibility from point A to point B; 3) Determine if the line from point A to point B ascends, descends, or is indeterminable; and 4) Select the line with the largest slope. Each test had four items of each of the four types of tasks, making it a sixteen-item test. The test items of the same type shared the same assignment and differed only in the map or the points marked on the map. An example of the test item is shown in Fig 2.

To assess the use of cognitive resources and the motivation of the users, two questionnaires were utilized. Both of them inquired about the beginning, middle, and ending phase of the educational training in the CIVE application. They consisted of statements, which were to be rated on a Likert-type scale. Users indicated their level of agreement by choosing an integer between one and seven, where one corresponded with “The statement does not fit at all” and seven corresponded with “The statement fits completely”. The first questionnaire incorporated statements about cognitive resources, which Wiley and Bailey [46] described as frames, long-term memory stores, and buffers. It inquired about perception, long-term memory, and short-term memory. The questionnaire was aimed at assessing how well did the single user or the dyad of users manage to: discover new information or perspectives; use the information they already knew; and keep important information while working with it. The second questionnaire was aimed at performance motivation and consisted of the items adapted from the scale of *flow* from the LMI inventory [47] by Schuler and Prochaska, in the Czech edition by Hoskovcová. The LMI is a performance motivation questionnaire, and the flow scale was used to assess if the user managed to work on a task intensively and with high concentration. The items were adapted to accommodate the circumstances of our research. Several examples of statements, rated in both questionnaires, are shown in Table 1.

**Table 1:** Examples of statements used in the questionnaires.

Questionnaire	Statement to be rated
Use of cognitive resources questionnaire	While working on the task, I/we managed to discover new information or ways of looking at the problem.
	While working on the task, I/we managed to use the information that I/we had discovered before.
	While working on the task, I/we managed to work with multiple pieces of information at the same time.
Performance motivation questionnaire	There were moments when I was fully focused on the task and everything else seemed unimportant to me.
	While working on the task, I felt satisfaction from the intensive and focused work.
	I completely immersed myself in the work on the task and the time passed incredibly fast.

**Fig 3:**
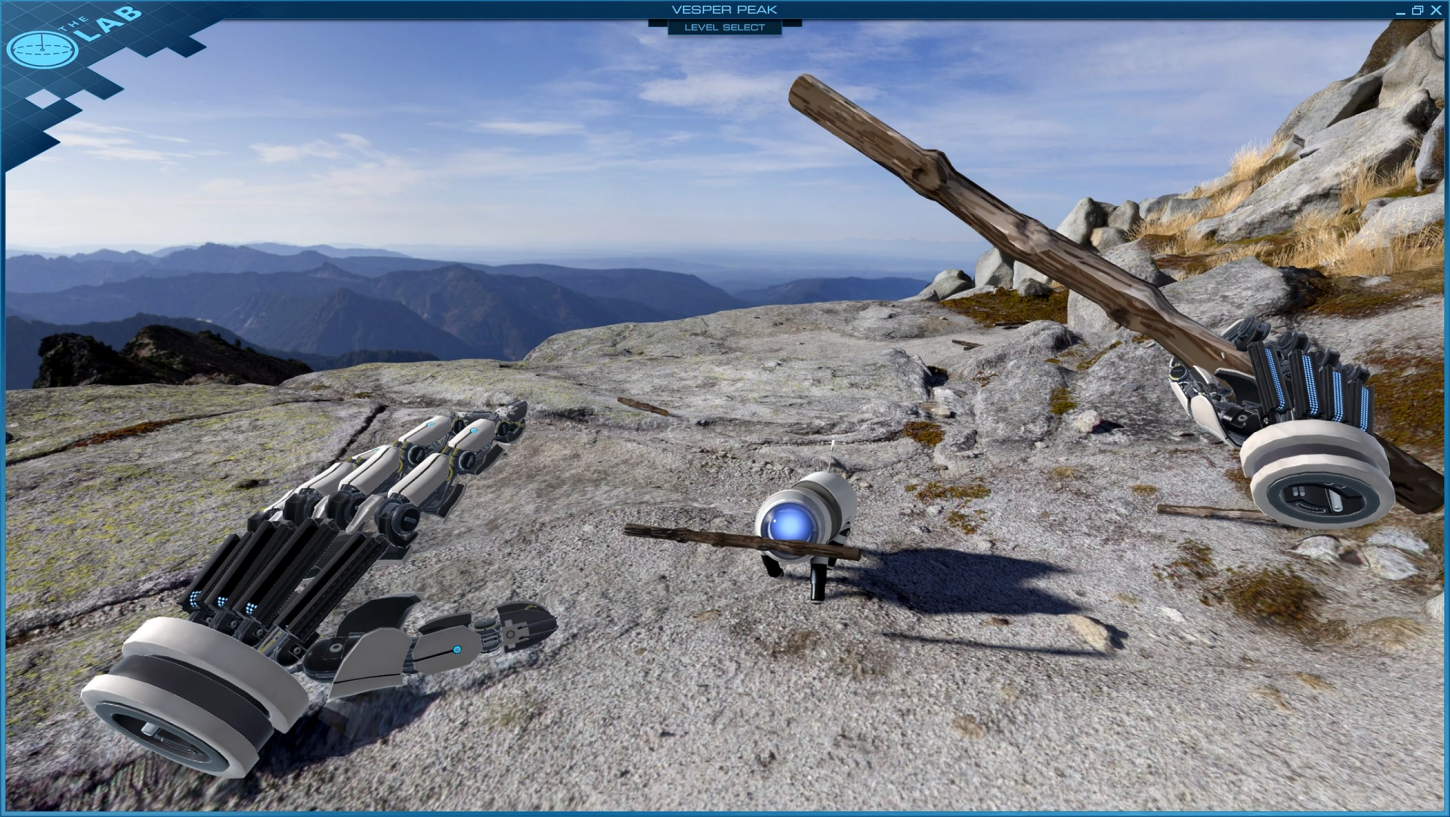
Virtual environment “Vesper Peak” used for iVR calibration and training.

For the purpose of adjusting the head-mounted display to the user and allowing them to get acquainted with the iVR controllers and movement in the virtual environment, a free-to-play application “The Lab” [48] was used. It was developed by Valve to showcase the possibilities of iVR. For example, in the environment “Vesper Peak”, the user is set on a mountain and can grab a stick from the ground to throw it to a mechanical dog, that will bring it back to the user, as shown in Fig 3.

### Research environment and conditions

Our research was conducted at the Faculty of Arts of Masaryk University in Brno, Czech Republic, in two rooms simultaneously. The first room was the HUME Lab Experimental Humanities Laboratory, and the second room was a lecture room on the same floor. There was a personal computer (Intel^®^ Core™ i7-5820K processor, Nvidia GeForce GTX 980 graphics card, 64 GB RAM) with an HTC Vive Pro full kit (1440 × 1600 pixels per eye, 90 Hz refresh rate, 110° field of view) connected to it in each room. The PCs were directly interconnected via a twisted-pair data cable running from one room to the other to minimize latency. The temperature and light in each room were adjusted to ideal conditions for iVR sensors’ operation and students’ performance. One participant and one research assistant were present in each room, with an additional supervising investigator for both rooms, to ensure the smooth flow of the procedure and to address potential unexpected technical issues.

### Participants

The participants of this research were recruited from a pool of volunteers consisting of students or absolvents of universities located in Brno. The inclusion criteria were age between 18 and 30, Czech or Slovak nationality, and regular use of PC. The exclusion criteria consisted of having a history of experiencing *cybersickness* [49] or attending any geography-related field of study at a university level. The objective of the criteria of the participants’ age and field of study was to allow for an investigation of the effectiveness of the CIVE application for peer collaboration. The final research sample of 80 participants had ages ranging from 19 to 29 years (mean age 22.325±2.288), 67.5% were female (*n* = 54) and 32.5% male (*n* = 26), and all reported daily use of PC. Participants were randomly divided into two experimental groups: individual learning in CIVE; and collaborative learning in CIVE.

### Ethics statement

The study was approved by the Research Ethics Committee of Masaryk University in Brno. Each participant signed an informed consent form prior to inclusion in the study. The confidentiality of personal information was strictly maintained, and the participants had the right to withdraw from the study at any time during the procedure.

### Procedure

**Fig 4:**
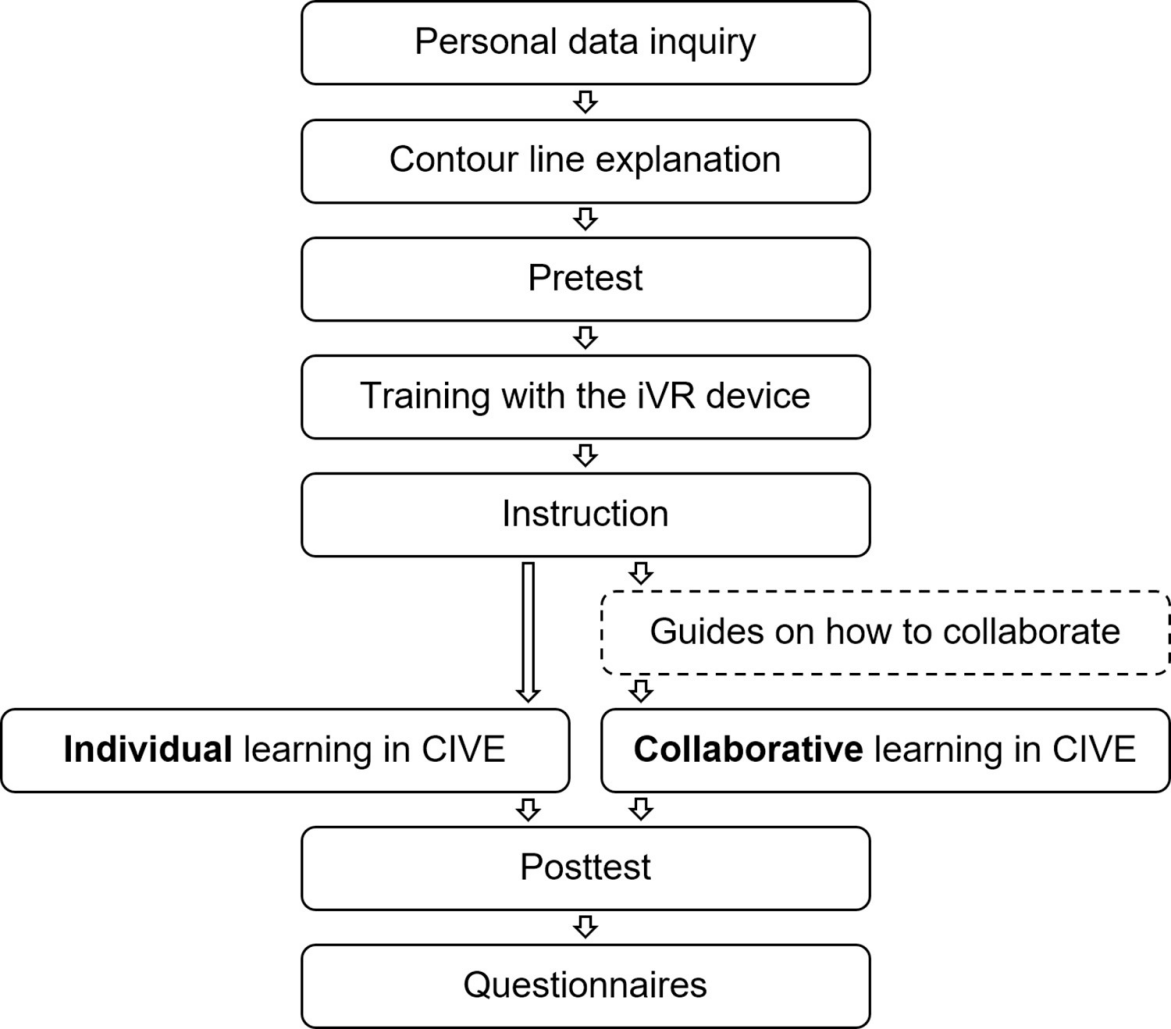
Overview of the research procedure flow.

The procedure differed for experimental groups in the circumstances of the immersive virtual learning intervention–whether the participant underwent it solo or in a dyad with another participant. The collaborative learning in CIVE group, therefore, received additional *Guides on how to collaborate* in the instructional step. The flow of the research procedure for both experimental groups is shown in Fig 4.

The research procedure consisted of eight steps: 1. Personal data inquiry; 2. Contour line explanation; 3. Pretest; 4. Training with the iVR device; 5. Instruction; 6. Learning in CIVE; 7. Posttest; and 8. Questionnaires. The first step involved *Personal data inquiry* and signing of the informed consent form. The information was collected in a verbal and written manner. That is, the participants filled out the form and were asked additional questions to clarify their written answers. They were inquired about their experience with iVR devices, non-iVR devices, and also about their education to ensure that they did not major in any geography-related field of study.

As the second step, there was a *Contour line explanation*. A definition of a contour line together with a basic explanatory picture thereof were given to the participants. This was done to ensure that all participants had knowledge about what a contour line looks like on a map, what it represents, and that the number subscribed to it refers to altitude. This was to ensure that the participants knew the meaning of the basic hypsographic element that was later used in the pretest, learning in CIVE, and posttest steps.

The third step was the *Pretest* consisting of four types of tasks, each with four test items. All tasks were related to the contour lines on a given map, however, required an ability to complexly analyze the map and infer a conclusion about the terrain it represents. Half of the participants in each experimental group received “geo α” as the pretest and “geo β” as the posttest, and half of the participants vice versa.

As the fourth step, the participants received *Training with the iVR device* in the form of a simple virtual experience using the “Vesper Peak” environment of Valve’s application “The Lab”. This served as screening against *cybersickness*. Participants were observed for signs of nausea and asked if they feel any discomfort. The headset was adjusted according to the size of the participant’s head and their interpupillary distance. Participants also had the opportunity to get acquainted with the iVR controllers and how to use them for interaction with an immersive virtual environment. The additional objective of this step was to mitigate the *novelty effect*, posing as a potential threat to the external validity of the research due to the effects that new technology has on its users [50].

The fifth step was *Instruction* and consisted of an explanation of the assignment and user interfaces for the subsequent task in the CIVE application. The collaborative learning in CIVE group received also additional *Guides on how to collaborate*. It was explained to them that they would see an avatar of their collaborator in the CIVE application and that they are supposed to work together on the given task. Verbal and non-verbal ways of communication were presented–participants could talk to each other via headphones and make gestures utilizing their avatars’ movements and positioning. In addition, virtual models of their iVR controllers, shown in the CIVE application, enabled the use of laser pointers. The dyad of participants was prompted to talk to each other, verbalize what they are currently working on, and discuss the solution before submitting it for evaluation.

The final three steps started with the sixth step–the participants underwent *Learning in CIVE* either solo or in a dyad, according to the experimental group they were assigned to. The duration of the learning intervention in our CIVE application was designed to be 30±5 minutes. The seventh step was the *Posttest* consisting of the same four types of tasks as the *Pretest*, but with different test items. Finally, as the eighth step, the *Questionnaires* were administered, aimed at motivation, use of cognitive resources, and evaluation of the learning procedure. The whole research procedure with each participant lasted approximately 90 minutes.

### Analysis

For quantitative data processing, IBM SPSS Statistics 27 was utilized. The independent variable had two levels–individual and collaborative learning. Each of the dependent variables was measured for both levels of the independent variable. Since the explored variables did not pass the *Shapiro–Wilk* test of normality (see Table 2) in half of the cases, nonparametric tests were chosen. Furthermore, since repeated measurements in two experimental groups were conducted, the *Friedman’s Two-way Analysis of Variance* and the *Mann–Whitney U test* were used to statistically analyze the distribution of values. A *p*-value under 0.05 was considered significant.

**Table 2:** Overview of the dependent variables.

Dependent variable	Levels of independent variables		Shapiro–Wilk test of normality		
			*W*	*df*	*p*
Geo-score	individual	pretest	0.942	40	0.041 *
		posttest	0.880	40	< 0.001 *
	collaborative	pretest	0.940	40	0.034 *
		posttest	0.846	40	< 0.001 *
Geo-speed	individual	pretest	0.966	40	0.274
		posttest	0.890	40	< 0.001 *
	collaborative	pretest	0.932	40	0.018 *
		posttest	0.962	40	0.203
Learning gain	individual		0.965	40	0.243
	collaborative		0.940	40	0.036 *
Speed gain	individual		0.911	40	0.004 *
	collaborative		0.972	40	0.413
Use of cognitive resources	individual		0.952	40	0.087
	collaborative		0.966	40	0.269
Performance motivation	individual		0.975	40	0.508
	collaborative		0.955	40	0.112

* *p*-value less than the significance level of 0.05.

To assess the effectiveness of individual and collaborative learning in CIVE for the education of geography, the Friedman’s Two-way ANOVA was applied separately on the data from each experimental group. Dependent variables representing *geo-score* and *geo-speed*, each having its pretest and posttest value, were processed. Changes in the distribution of variable values between repeated measurements were evaluated to determine whether the degree of change in geo-score and geo-speed was significant. Additionally, to test whether the collaborative use was more effective, the *Gain Score Approach* [51] was utilized. The Gain Score of geo-score was calculated as the pretest value subtracted from the posttest value and was labeled *learning gain*. The Gain Score of geo-speed was calculated as the pretest value subtracted from the posttest value and was labeled *speed gain*. Subsequently, the Mann–Whitney U test was used to compare the learning gain and speed gain between the experimental group of individual and collaborative learning in CIVE. Based on a comparison of the distribution of values in these two groups, the significance of the difference was calculated. The Mann–Whitney U test was also used to investigate the difference in the *use of cognitive resources* and the *performance motivation* of participants between the experimental groups. The dataset used for descriptive statistics and inductive statistical analysis is presented in S1 Table.

## Results

**Fig 5:**
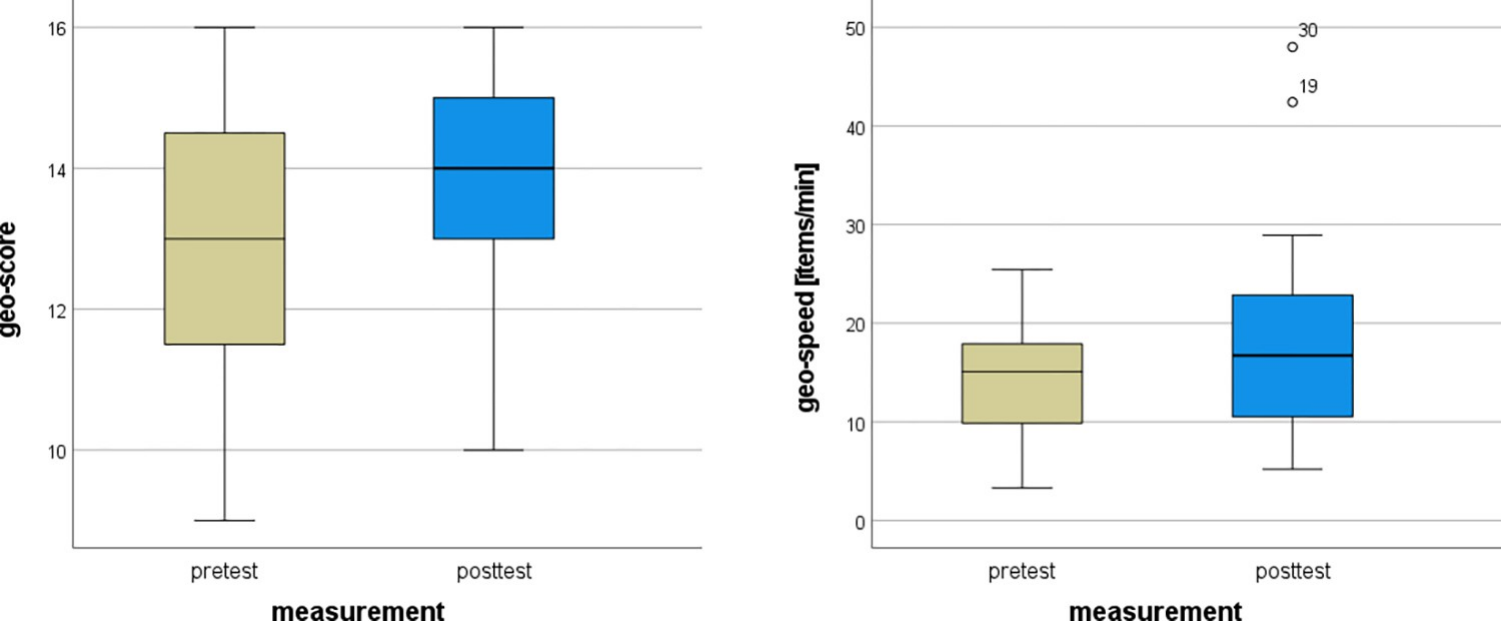
Pretest and posttest values for score and speed in geo tests for individual learning.

The Friedman’s Two-way ANOVA was applied to the individual learning experimental group data from the geographical pretest and posttest. The results showed that there was a statistically significant difference in both the geo-score (*Fr* = 5.121, *df* = 1, *p* = 0.024, *ES* = 0.128) and the geo-speed (*Fr* = 4.900, *df* = 1, *p* = 0.027, *ES* = 0.123). Individual learning in CIVE was effective in increasing the score in the geographical test and increasing the speed of solving. The distribution of the values is shown in Fig 5. The mean geo-scores in the pretest and posttest were 13.025±1.847 and 13.800±1.897, respectively, while the mean geo-speeds in the pretest and posttest were 14.023±5.476 and 17.486±8.986 items/min, respectively.

**Fig 6:**
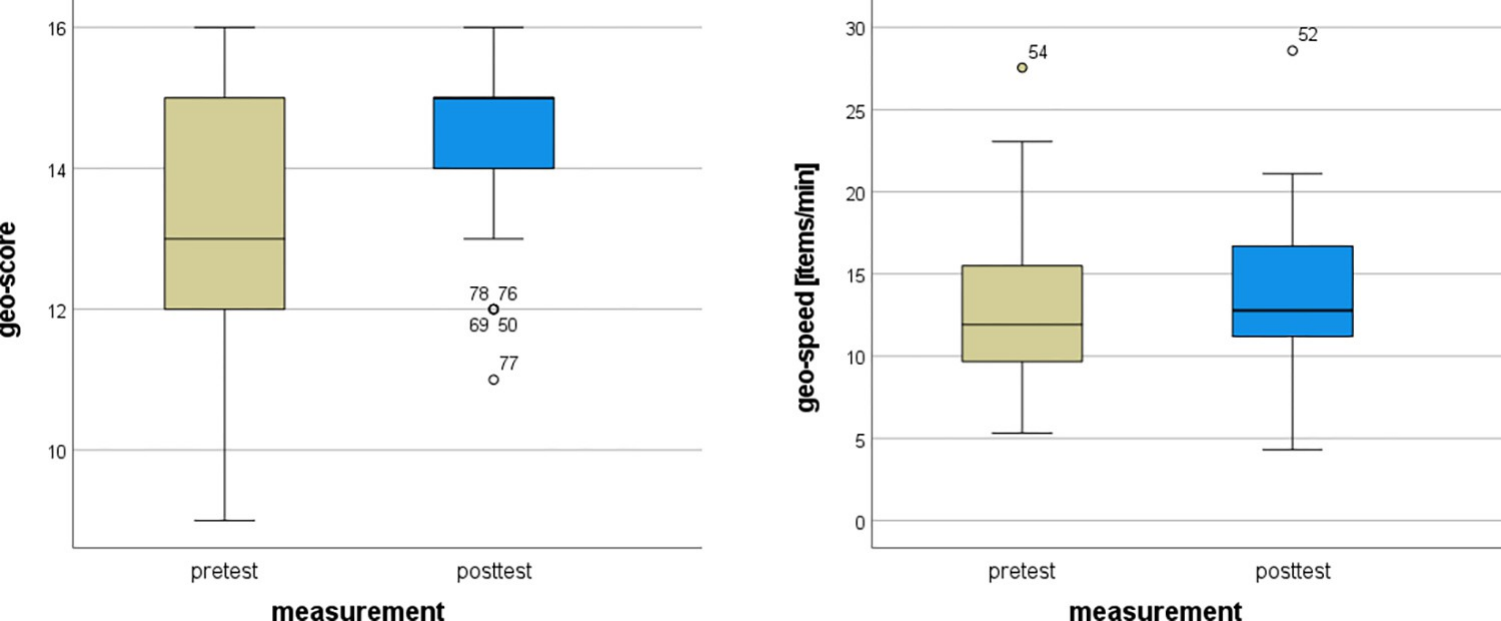
Pretest and posttest values for score and speed in geo tests for collaborative learning.

Using the Friedman’s Two-way ANOVA on the collaborative learning experimental group data from the geographical pretest and posttest, we obtained the following results. There was a statistically significant difference in the geo-score (*Fr* = 13.333, *df* = 1, *p* < 0.001, *ES* = 0.333) but not in the geo-speed (*Fr* = 0.000, *df* = 1, *p* = 1.000, *ES* = 0.000). Collaborative learning in CIVE was effective in increasing the score in the geographical test; however, the increase in the speed of solving was not significant. The distribution of the values is shown in Fig 6. The mean geo-scores in the pretest and posttest were 13.125±1.964 and 14.375±1.254, respectively, while the mean geo-speeds in the pretest and posttest were 12.504±4.586 and 13.629±4.868 items/min, respectively.

**Fig 7:**
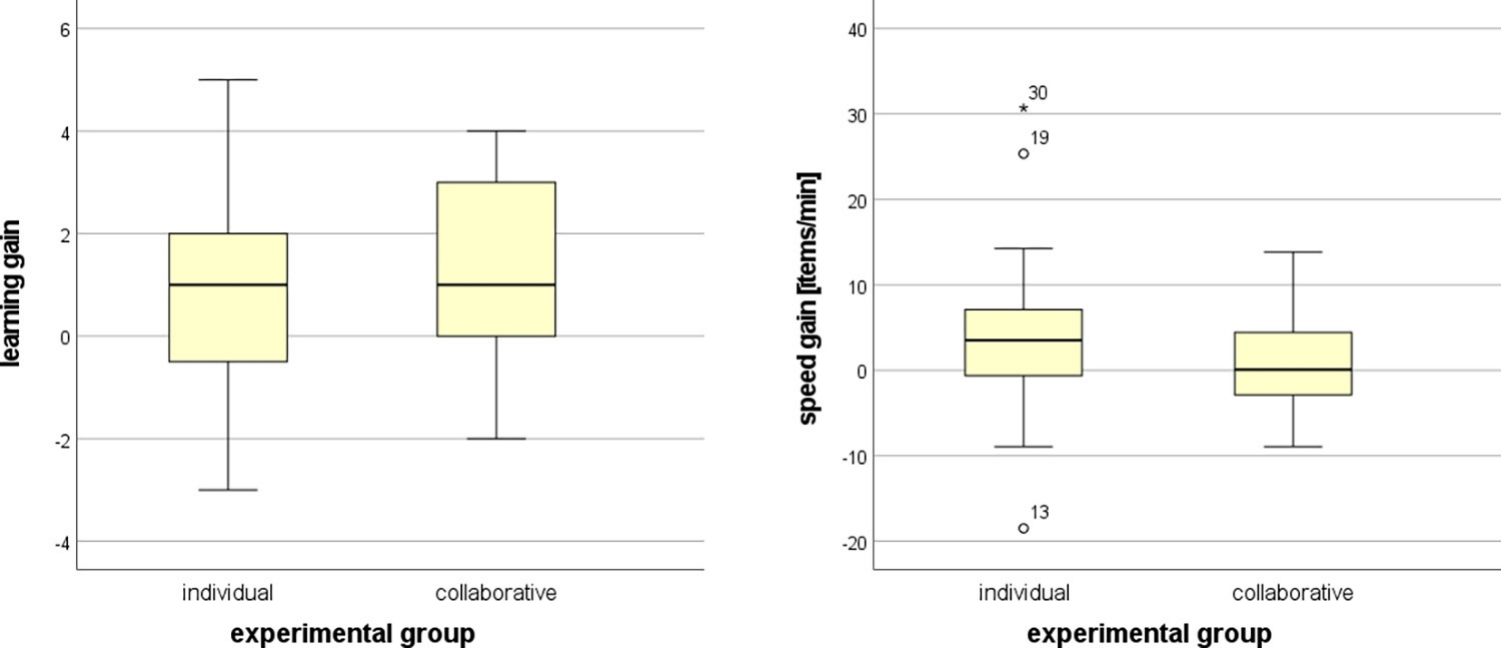
Comparison of learning gain and speed gain between experimental groups.

The Mann–Whitney U test was utilized to compare the learning gain and the speed gain between the experimental group of individual and collaborative learning in CIVE. The results showed that there was no statistically significant difference between the learning gains (*U* = 905.5, *p* = 0.303, *ES* = 0.115), nor between the speed gains (*U* = 628.0, *p* = 0.098, *ES* = 0.185). We hypothesized that collaborative learning would be more effective; however, the data was inconclusive and did not confirm this. Even though significant evidence was not found, the results indicated the collaborative learning group to be more effective in achieving higher learning gain but lower speed gain in comparison to the individual learning group. The distribution of the values is shown in Fig 7. The mean learning gains in the individual and collaborative groups were 0.775±1.915 and 1.250±1.581, respectively, while the mean speed gains in the individual and collaborative groups were 3.463±8.349 and 1.125±5.312 items/min, respectively.

**Fig 8:**
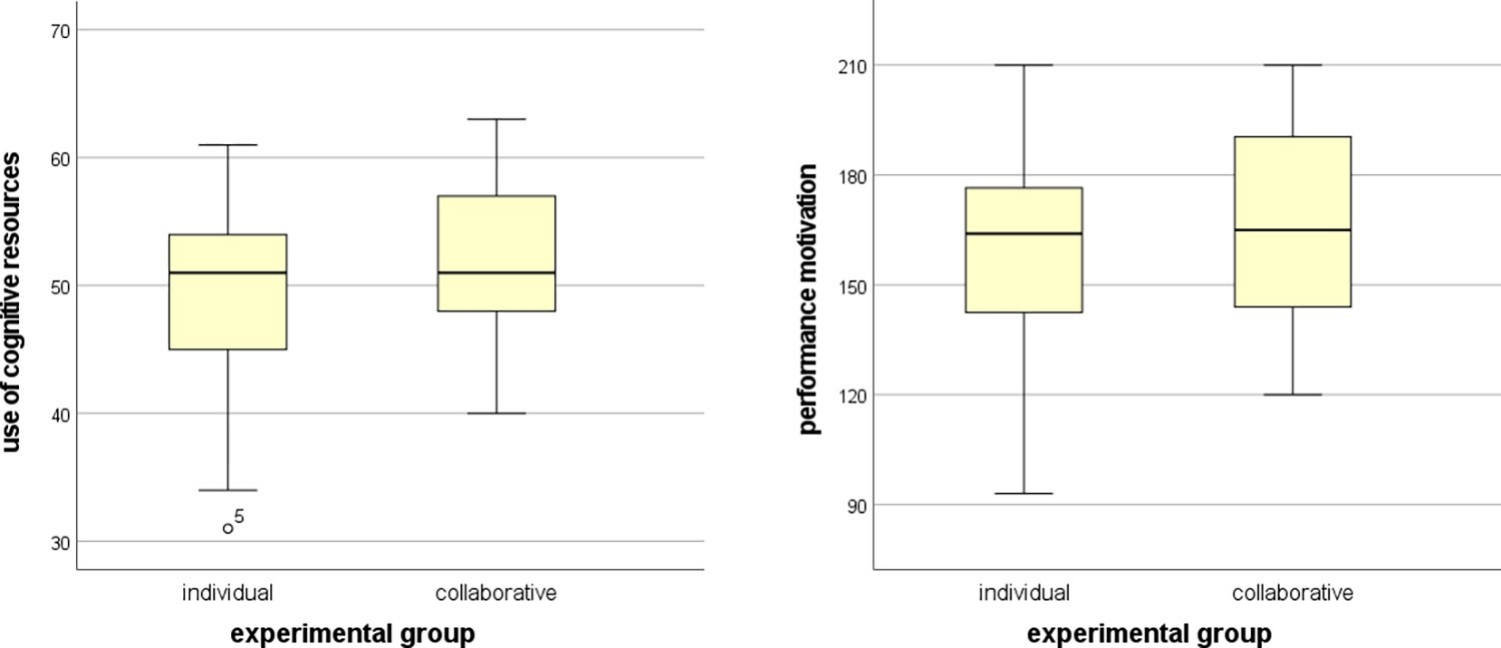
Comparison of use of cognitive resources and motivation between experimental groups.

Using the Mann–Whitney U test on the scores for the use of cognitive resources and the performance motivation of the participants, we compared the experimental groups and obtained the following results. There was a statistically significant difference in the use of cognitive resources (*U* = 1007.0, *p* = 0.046, *ES* = 0.223) but not in the performance motivation (*U* = 893.5, *p* = 0.368, *ES* = 0.101). The results showed that the collaborative learning group achieved significantly higher scores in the use of cognitive resources questionnaire; however, the increase in scores in the performance motivation questionnaire was not significant, in comparison to the individual learning group. The distribution of the values is shown in Fig 8. Finally, the mean use of cognitive resources in the individual and collaborative groups were 48.650±7.109 and 52.225±5.673, respectively, while the mean performance motivation in the individual and collaborative groups were 159.350±26.331 and 165.575±25.689, respectively.

## Discussion

The main objective of this study was to assess the effectiveness of learning geography in the immersive virtual environment in terms of both individual and collaborative use. Further objectives included comparing the individual and collaborative use in terms of the effectiveness, use of resources, and motivation. We conducted the study utilizing our CIVE application for hypsography education, developed at Masaryk University. Eighty participants underwent the training intervention in the application either individually or in dyads.

The individual learning in CIVE was shown to be effective, both in increasing the score and the speed of solving geospatial tasks. Similar results showing the usefulness of iVR for complex skill acquisition can be found, for example, in the education of orthopedy [52], electrical circuitry [53], laboratory safety [9], mathematical problem-solving [54], and surgical skills [55].

The collaborative learning in CIVE was shown to be effective in increasing the score; however, not in increasing the speed of solving geographical tasks, as there was in the case of the individual learning. It can be argued that this unexpected result can be attributed to the *cognitive conflict* that arose from collaboration. According to Piaget’s [56] theory of cognitive development, a student in a learning process may experience a state of *disequilibrium* created by a collaborator. This cognitive conflict fuels the tendency to re-establish equilibrium through the process of adaptation and modification of the student’s cognitive structures. Argumentation with a collaborator may have resulted in a broader range of alternatives, which the student then considers while solving the geographical task independently, hence taking them more time to choose an answer.

Another aim of this study was to compare the effectiveness of individual and collaborative learning of geography in CIVE. We hypothesized that the collaborative use would be more effective, yet the results did not show a significant difference. In the literature comparing individual and collaborative learning, there has been ambivalence about which one is more effective. Several studies have concluded that collaborative learners achieve higher performance in the learning process than individual learners–⁠ for example, the study by Okada and Simon [57] aimed at scientific discovery learning, and the study by Teasley [58] aimed at scientific reasoning tasks. However, Fawcett and Garton [59] also reported significantly higher performance in the learning process by dyads, but the learning gain calculated from the pretest and posttest did not show a significant difference between the collaborative and individual learning groups. It should be noted that in a study of individual versus collaborative learning, the context of the experiment tends to be of particular importance. For example, the study of the collective working memory [60] in the domain of biology learning showed that the complexity of the task had an effect on the learning process efficiency. The collaborative learning was more efficient with complex tasks, while the individual learning had bigger efficiency with low-complexity tasks.

The last objective of this study was to investigate the difference in the use of cognitive resources and the motivation of participants who underwent the geography learning CIVE intervention individually and collaboratively. The results for the difference in performance motivation were inconclusive. The performance motivation LMI inventory’s [47] scale of flow was utilized to measure the degree of engagement in the task. Csikszentmihalyi [38, 39], the author of the concept of flow, described it as intense engagement and concentration on a task, which result in high productivity and positive emotions. The achieved flow was high in both experimental groups, and although the scores were overall higher in the collaborative group, the results did not show a statistical difference between them.

On the other hand, collaborative learning facilitated significantly higher use of cognitive resources than individual learning, in the sense of the amount of information the participants were able to discover, process, and remember–⁠ as described by Wiley and Bailey [46]. The collaborative participants of our study reported it was easier for them to discover new information or perspectives during the process of solving the geographical task; to keep important information in the short-term memory; and to recall important information from the long-term memory. The presence of a collaborator enabled them to pool their cognitive resources to collaboratively process more information. However, this has not occurred in all collaborative learning studies, because the cognitive load in collaborative tasks tends to be higher, as collaborators need to process information from each other, create responses, and socially interact. This additional cognitive load is referred to by Dillenbourg and Bétrancourt as a *collaboration load* [61]. The participants in our previous study [20] described they lacked facial expressions in the avatars’ faces. However, it can be argued that this could have been one of the factors that helped to decrease the collaboration load in our CIVE application, and that participants did not have to process this information in addition to the task-related information. A future study aimed at this hypothesis would be appropriate. Kolfschoten and Brazier [62] stressed the importance of a thoughtful design of the collaborative task, as well as the need to address the problem of cognitive load. The task and scaffolding should lead the collaborators to the *convergence*–that is, to the creation of an overview of perspectives and alternatives that the collaborators are considering.

Our previous qualitative study [20] uncovered themes that were important for students in the CIVE application. Participants reported perceiving 3D terrain representations in iVR as useful for learning geography. They also appreciated the presence of a collaborator for brainstorming ideas and verifying task solutions before submitting them. The participants expressed they would have felt lost without a collaborator. These themes indicated that iVR could be effective for learning geography and that collaborative use could be beneficial for students. However, the results were based on subjective statements acquired through semi-structured interviews. The present quantitative study confirmed that iVR as a medium is an effective tool for learning skills that require spatial representations. It offers an alternative to classical web-based learning and can be utilized, for example, in cases of social distancing or lockdowns. We also wanted to show that collaborative learning in iVR would be more effective than individual learning, but this was not achieved. However, we theorize that collaborative use of iVR might be more effective in didactical scenarios that are either more complex or require the application of multiple concepts, for example, ecological topics or the water cycle in nature. This outlines the main limitation of the study. The generalizability of the results may be limited due to the diversity that can be found in pedagogy, educational scenarios, and instructional approaches. In future research, it would be desirable to explore and compare more types of learning in iVR, such as tutoring by a teacher or group learning. It would be appropriate to draw generalized conclusions from a meta-analysis of a broader knowledge base obtained from a number of individual studies such as this one.

## Conclusion

This study investigated the effectiveness of the collaborative immersive virtual environment, which our interdisciplinary team developed, for learning and training in geography. The results showed that the individual use of the application significantly increased scores and speed in hypsography tests, and the collaborative use significantly increased scores. The speed in hypsography tests did not increase after collaborative learning. This was discussed in relation to cognitive and social factors, such as additional cognitive load generated from collaboration, and the internalization of alternatives and perspectives obtained from discussion with a collaborator in the learning process. In general, iVR as a medium has been shown to be an effective tool for learning geography, regardless of the instructional approach used. Additionally, to compare collaborative and individual learning approaches, we calculated learning gain and speed gain for both experimental groups. However, their statistical comparison produced inconclusive results, only indicating that the collaborative learning group achieved higher learning gain. Furthermore, we compared the use of cognitive resources and performance motivation between the experimental groups. The difference in motivation was not significant; however, the results showed significantly higher use of cognitive resources in the collaborative group. Further investigations comparing individual and collaborative learning in immersive virtual reality were, therefore, proposed.

## Supporting information

S1 Table Dataset used for descriptive statistics and inductive statistical analysis. (SAV)
